# Pairwise ratio-based differential abundance analysis of infant microbiome 16S sequencing data

**DOI:** 10.1093/nargab/lqad001

**Published:** 2023-01-20

**Authors:** Kevin Mildau, Dennis E te Beest, Bas Engel, Gerrit Gort, Jolanda Lambert, Sophie H N Swinkels, Fred A van Eeuwijk

**Affiliations:** Biometris, Wageningen University & Research, 6700 HB Wageningen, The Netherlands; Biometris, Wageningen University & Research, 6700 HB Wageningen, The Netherlands; Biometris, Wageningen University & Research, 6700 HB Wageningen, The Netherlands; Biometris, Wageningen University & Research, 6700 HB Wageningen, The Netherlands; Danone Nutricia Research, Uppsalalaan 12, 3584 CT Utrecht, The Netherlands; Danone Nutricia Research, Uppsalalaan 12, 3584 CT Utrecht, The Netherlands; Biometris, Wageningen University & Research, 6700 HB Wageningen, The Netherlands

## Abstract

Differential abundance analysis of infant 16S microbial sequencing data is complicated by challenging data properties, including high sparsity, extreme dispersion and the relative nature of the information contained within the data. In this study, we propose a pairwise ratio analysis that uses the compositional data analysis principle of subcompositional coherence and merges it with a beta-binomial regression model. The resulting method provides a flexible and easily interpretable approach to infant 16S sequencing data differential abundance analysis that does not require zero imputation. We evaluate the proposed method using infant 16S data from clinical trials and demonstrate that the proposed method has the power to detect differences, and demonstrate how its results can be used to gain insights. We further evaluate the method using data-inspired simulations and compare its power against related methods. Our results indicate that power is high for pairwise differential abundance analysis of taxon pairs that have a large abundance. In contrast, results for sparse taxon pairs show a decrease in power and substantial variability in method performance. While our method shows promising performance on well-measured subcompositions, we advise strong filtering steps in order to avoid excessive numbers of underpowered comparisons in practical applications.

## INTRODUCTION

The interest in the early life development of the human microbiome has grown substantially in recent years, as have publications showcasing some form of differential abundance analyses (1,2). Microbiota compositions are measured using culture-independent techniques such as 16S metagenomic sequencing with the aim of finding associations between microbiota compositions and diseases or to assess the impact of interventions on composition (3). The statistical analysis of infant 16S sequencing datasets is challenging due to a combination of data properties. These data are extremely sparse, they have a high dispersion and they are compositional (4–7). Sparsity and dispersion are the result of heterogeneity among experimental units (i.e. infants and technical variation). Infant microbiome data are characterized as hypervariable, developing microbial ecosystems for which both compositions and absolute abundances tend to fluctuate widely across experimental conditions or time (8). The compositionality is a consequence of the fact that the sequencing depth is a technical artifact that is not informative of the underlying total abundances, and of the lack of internal standardization in the measurement approach (5,9–13).

Many commonly applied data analysis approaches deal with compositionality through some form of data standardization, commonly referred to as ‘normalization methods’, that assumes the existence of some stable features shared across all samples (14,15). While such assumptions are commonly accepted in some RNA-seq contexts (16–18) and may apply to various stable microbial ecosystems, they are inappropriate for infant microbiomes. The lacking applicability of standardization techniques and the extreme variability of infant compositions call for the development of appropriate methodology for differential abundance analysis.

Beta-binomial models have a long history of being applied to overdispersed count data to be analyzed as proportions (19–22), and variants or extensions of the model have been suggested for microbiome data analysis (23,24). The method proposed by Martin *et al.* (23) models the proportions of each taxon with respect to all other taxa, which is conceptually the same as modeling the data as relative abundances (25–28). This approach uses the totals as a normalization, which can be appropriate when total loads stay constant across samples and conditions, in which case changes in relative abundance correspond to changes in absolute abundances up to a scaling constant (15). While this approach is compositional in the sense that it respects the unit-sum constraint of the data, it suffers from interpretational shortcomings well known in the compositional data analysis field. Specifically, all relative abundance techniques are affected by what is referred to as the ‘compositional’ or ‘closure’ bias, i.e. the feature of compositional data that forces all proportions to change when only one taxon actually changes in the absolute sense (5,29,30). The dependence of each proportion on all others through closure alone hampers interpretation of univariate tests based on them. Moreover, since all other taxa are amalgamated into one reference in relative abundance analyses, it becomes difficult to compare differential relative abundances across studies or measurement platforms with more limited taxonomic coverage. While it is impossible to perform absolute abundance analyses on the data without additional assumptions, it is possible to make precise inferences by freeing analyses of closure bias by focusing on ratios of taxa (31,32). For these reasons, we propose merging the pairwise ratio perspective with the beta-binomial regression model. Instead of modeling proportions relative to the total library size, we condition on the pairwise totals and thus model proportions relative to the pairwise totals. As a result, we create a flexible method that provides inferences without requiring standardization, does not need zero imputation and leads to results that are subcompositionally coherent and thus comparable across studies.

We evaluate the proposed method with an application and a simulation study. In the application section, we compare our method to the beta-binomial model proposed by Martin *et al.* (23). In the latter, we evaluate the method in terms of type I error rates and power for realistic *in silico* 16S sequencing data inspired by infant microbiome data from clinical trials. We compare with alternative methods that model pairwise ratios to gain further insights into the performance of the proposed method. The remainder of this paper is structured as follows. In the ‘Materials and Methods’ section, we first describe the pairwise beta-binomial regression model. Next, we describe some additional methodological aspects concerning multiple comparisons and the interpretation of pairwise results. We then describe the infant reference data used for evaluation of the method and outline the application and the simulation study. In the ‘Results’ section, we describe the findings of the application and the simulation study. In the last section, we discuss the scope and limitations of our results and give recommendations for scientists involved in the analysis of infant microbiome data.

## MATERIALS AND METHODS

### Pairwise beta-binomial generalized linear model

The focus of the proposed method is on the univariate analysis of one taxon pair using the beta-binomial model. We model the abundance as a pairwise ratio, i.e. one taxon with respect to another taxon. Indexing two taxa by *a* and *b*, and indexing the samples by *i*, the beta-binomial model can be denoted by

\begin{eqnarray*} Y_{i,a} |(P_i, T_{i,ab}) \sim {\rm Binomial}(T_{i,ab}, P_i), \end{eqnarray*}

**Figure 1.**
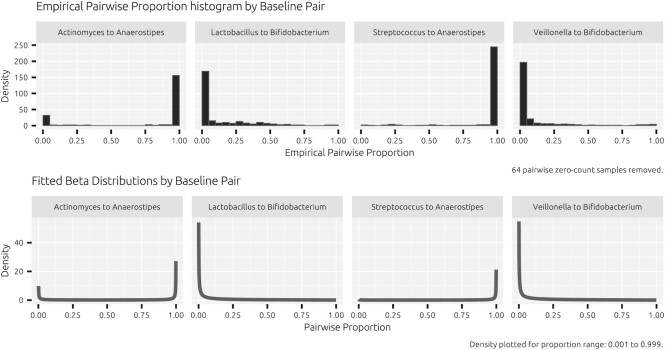
Histograms of pairwise proportion (${Y_{a}}/{T_{ab}}$) for four selected taxon pairs and corresponding fitted beta distributions of the beta-binomial model. Raw data proportions show extreme dispersion and sparsity. In many samples, either one of the taxa has a zero or close to zero count, represented by large fractions of proportions close to or at the boundaries of the proportional spectrum. For some taxon pairs, both taxa may be registered as a zero count, making it impossible to establish pairwise proportions or ratios. The beta component of the beta-binomial models shows adequate flexibility to account for observed distributional patterns.

\begin{eqnarray*} P_i \sim {\rm Beta}(\alpha _{1,i} , \alpha _{2,i}), \end{eqnarray*}

where *Y*_*a*_ denotes the count of taxon *a* and *T*_*ab*_ denotes the total count of taxa *a* and *b*. The count of taxon *b* is implicit in the model via the pairwise totals *T*_*ab*_, where *Y*_*b*_ = *T*_*ab*_ − *Y*_*a*_. Sample-specific binomial probabilities *P*_*i*_ are assumed to follow a beta distribution with parameters α_1,*i*_ and α_2,*i*_. In this model, the (latent) beta distribution describes the shape of the pairwise relative abundance per taxon pair; the beta distribution is flexible and commonly used to model proportions (see Figure 1 for an example). The binomial part models the accuracy with which the pair is observed in the data. The beta distribution has an expected pairwise relative abundance given by $E(P_i) = \mu _i = {\alpha _{1,i}}/ ({\alpha _{1,i} + \alpha _{2,i}} )$ and a dispersion given by $\theta _i = {1}/ ({\alpha _{1,i} + \alpha _{2,i}})$. From here we can model the probability *μ* with a linear predictor using the logit link, i.e. ${\rm logit}(\mu _i) = {\rm log}[{\mu _i}/ ({1-\mu _i} )] = \pi _i$. Here, *μ*_*i*_ and 1 − *μ*_*i*_ describe, respectively, the relative abundance of taxon *A* and taxon *B* relative to the pairwise total. To make the connection to pairwise ratios more evident, note that ${Y_{a}}/{T_{ab}} = [1 + ({Y_{b}}/{Y_{a}})]^{-1}$. The linear predictor (*π*_*i*_) may contain any qualitative or quantitative explanatory variable. When comparing expected pairwise relative abundances across two treatment groups, we can set *π*_*i*_ = *β*_0_ + *β*_1_*x*_*i*_. For a two-group comparison, the parameter *β*_0_ represents the log odds of taxon *a* with respect to taxon *b* in the control group. The parameter *β*_1_ describes the difference in log odds between the treatment and control groups, indicated via the treatment dummy variable *x*_*i*_. After exponentiation, *β*_1_ represents the odds ratio of taxon *a* with respect to taxon *b* across treatment and control.

A recent paper describes how count data contain scale information, and that small counts can be an issue (33). In our proposed method, we model the amount of information in the data with the binomial part. For a taxon pair that contains a large number of small counts, we can less accurately estimate the pairwise relative abundances, which will lead to larger confidence intervals and a low power, which is an accurate reflection of the amount of information available. Note that the pairwise totals are affected by the sequencing depth: a higher depth means a higher pairwise total, and vice versa. For moderate pairwise totals, the variance of the beta-binomial is approximately equivalent to the variance of the often sizable beta component, i.e. ${\rm Var}( {Y_{i,a}}/{T_{i, ab}}) = [({1}/{T_{i,ab}}) + ({(T_{i,ab}-1)}/{T_{i,ab}})\phi ]\mu _a(1-\mu _a)$. If *T*_*ab*_ is large, we have ${\rm Var}(Y_{i,a}/T_{i,ab}) \simeq \phi \mu _a(1-\mu _a) = [{\theta }/{(1+\theta ) ]}\mu _a(1-\mu _a)$, where *ϕ* models extra-binomial variation. The latter term is equivalent to the variance of the beta distribution. This means the latent beta distribution variability is expected to dominate over binomial count variability with moderate or large pairwise totals.

### Software

The pairwise beta-binomial model can be fitted with any beta-binomial implementation. A number of implementations exist in R, most notably corncob (23), glmmTMB (34) and gamlss (35). Each of these has its own advantages and collectively they contain a large statistical toolbox that can be used in combination with a pairwise beta-binomial model. In this paper, we only use corncob because it has a speed advantage over the mentioned alternatives, which is useful in a simulation study or doing a full pairwise comparison. Note that for practical applications speed is unlikely an issue with any of the mentioned implementations, as a single model fits in a matter of seconds. For a basic code example in R, we refer to Supplement A. The code used in the analyses is included in Supplement D.

### Multiple comparison

Each taxon pair can be analyzed separately, which means that the number of possible comparisons that can be made increases quadratically with the number of evaluated taxa. With *k* taxa, there are $({1}/{2})(k^2-k)$ possible ratios. A multiple comparison correction across all tested ratios is thus required. In this data example, we apply the Benjamini–Hochberg method (36) across all tested ratios. Benjamini–Hochberg is computationally quick to apply, robust and frequently applied with omics data to calculate the false discovery rate (FDR).

### Selecting taxa

Although the inference with the pairwise beta-binomial model is on the level of the ratio, the interest may still be in identifying a subset of taxa. This requires some strategy on how to interpret the pairwise results. As a first step, it is useful to examine a visualization of a pairwise result (as example see Figure 3). This figure displays all ratios on the *x* and *y* axes and each square in the figure represents one ratio. The blue squares represent significant ratios.

**Figure 2.**
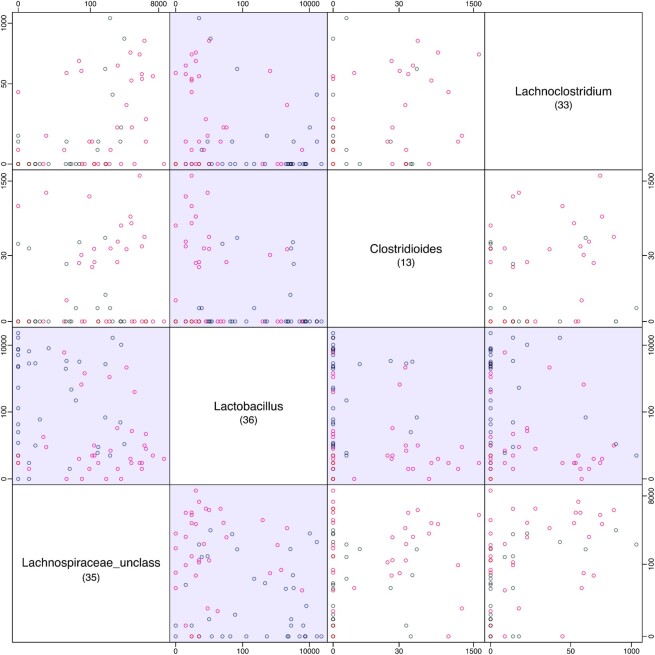
A visualization of a small subset of taxa of the data example. Displayed are four taxa plotted against each other that correspond to the bottom left 4 × 4 taxa of the treatment 2 versus treatment 4 comparison of Figure 3 (as indicated by the corresponding numbers). The blue coloring corresponds with Figure 3 and indicates that the null hypothesis (treatment 2 versus treatment 4) was rejected for that ratio. This figure illustrates the high variability and large numbers of zeros encountered with infant microbiome data. The data are log transformed using a pseudocount of 1.

**Figure 3.**
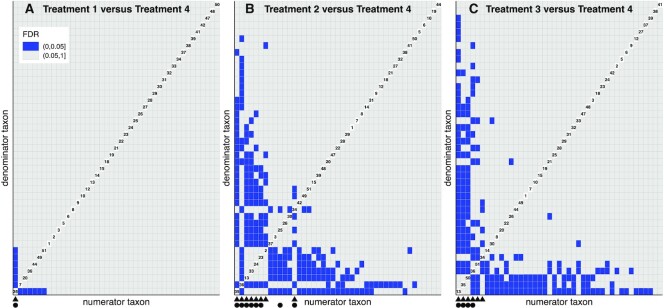
Visualization of the result with treatment 1 (**A**), treatment 2 (**B**) and treatment 3 (**C**) versus treatment 4. Each square represents a ratio between two taxa that is tested with the pairwise beta-binomial model. The diagonal contains numbers that indicate which taxa are used in a particular ratio; these numbers can be connected to taxonomy. Significant ratios [classified with FDR(BH) < 0.05] are colored in blue; ratios >0.05 are colored gray. Only taxa with at least one significant ratio in any of three comparisons have been included in the grid. Note that the grid is symmetric. The triangles at the *x*-axis indicate taxa that together contain most of the significant ratios in the pairwise beta-binomial model. The black circles at the *x*-axis indicate the taxa that are identified by the MWW method [FDR(BH) < 0.05].

One possibility is then to assign each ratio to one taxon, and then find a set of taxa that together are involved in most or all of the significant ratios. One way of identifying such a set is by ‘unwrapping’ the ratios. In this approach, we first select the taxon that has the most significant ratios. We then exclude all ratios this taxon is involved in, and select the taxon that is next involved in the most (remaining) significant ratios, and so on, until all significant ratios are accounted for. The resulting set of taxa can then be selected as interesting for further investigation. The order in which we unwrap the ratios provides a ranking.

A second possibility is to simply count the number of significant ratios each taxon is involved in, and use this as a ranking. The top of this ranking will often be similar to that of the first approach. Because here each (significant) ratio is assigned to two taxa, more ratios are assigned to seemingly unimportant taxa, which makes a stringent selection more difficult. Note that the ratio count of the second option is similar to the statistic (‘*W*’) that is used in method called ANCOM (37), which also attempts to identify taxa from ratio results, albeit with a very different approach.

Irrespectively of the selection method, taxa that are involved in many significant ratios may have a shift in their underlying abundance. Note that, given the compositional nature of the data, there is no guarantee that being involved in many significant ratios corresponds to underlying actual shifts. Similarly, it is possible that taxa with absolute shift do not have significant ratios (i.e. when all taxa shift in the same direction). Both issues are inherent to compositional data, and not related to the method. Also note that we do not classify individual taxa as significant or nonsignificant, because such statements are not possible with a pairwise beta-binomial model (i.e. it analyzes ratios).

### Reference data

The proposed method is evaluated using a subset of 16S sequencing data extracted from two infant clinical trials where the impact of various infant formulas on the gut microbiome was studied (clinical trial references: https://trialsearch.who.int/?TrialID=NTR2521 and https://trialsearch.who.int/?TrialID=NTR3455). In both studies, highly similar infant formulas with and without fermented components are compared in a randomized, double-blind clinical trial. In each study, 16S sequencing workflows provide insights into broad compositional trends in the data. Both clinical trial datasets were processed jointly (see details in Supplement B). Based on available metadata, a selection of samples based on infant age at sampling time has been made, restricting the age of all used infant samples to 3.5–6.5 months. If technical replicate samples were available for an infant, these were removed to ensure one sample per infant. The data of a selection of taxa are displayed against each other in Figure 2. The data are available in Supplement D.

### Evaluation

The evaluation is done by applying the method to an example from the reference data and with a data-driven simulation study. In the application, we compare the result of the proposed method to the closely related beta-binomial model of Martin *et al.* (23); we will refer to this method as the MWW method.

In the simulation study, we further evaluate the robustness and performance of the pairwise beta-binomial regression model and compare it to competing pairwise methods. The application is based upon one study (149 samples) and in the simulation study we use all 272 samples of both studies. In both parts of the evaluation, we use a set of 51 taxa (genus level) that were prevalent in at least 20% of the samples; any taxa at lower prevalence were removed. In both parts of the evaluation, testing is done using the null hypothesis of *H*_0_: *β*_1_ = 0 with *H*_a_: *β*_1_ ≠ 0 using a likelihood ratio test (38). Both model fitting and likelihood ratio testing are done using corncob (23); 95% confidence intervals are created using approximate normality assumptions of the sampling distribution of the parameter and the estimated standard error provided by corncob via $\hat{\beta }_1 \pm 1.96 \times {\rm se}(\hat{\beta }_1)$ (38).

### Application

In the application, we compare three different types of infant formulas (sample sizes of, respectively, 34, 37 and 42 infants, named treatments 1–3) separately against one reference formula (sample size of 36, named treatment 4). The research question is which (ratios of) taxa have changed in the infant microbiome as a result of being given treatment 1, 2 or 3 versus being given treatment 4. The taxa/ratios that are identified may provide further insights into how the feeds differ in their biological working. In total, this gives three different treatment comparisons. In this example, we analyze all combinations between 51 taxa (giving 1275 pairs). Per treatment comparison, we adjust for multiple comparisons across all ratios by calculating the FDR using Benjamini–Hochberg. Next, we classify as significant all ratios that have a FDR below 0.05; here, in practice a different threshold can be chosen. We visualize the rejected ratios with a grid that gives an overview of how findings per taxon pair/ratio relate to the individual taxa (as example see Figure 3). An example of a small subset of ratios with the data is displayed in Figure 2.

Next, we identify a limited set of taxa that are involved in the significant ratios (using the earlier described method). Finally, we make a comparison with the MWW method (23). Although the focus of this method is different (i.e. individual taxa versus ratios), we can compare the sets of taxa that are identified. With the MWW method, we test each taxon and calculate the FDR (with Benjamini–Hochberg) across the taxa. We then classify taxa with an FDR below 0.05 as significant.

### Simulation study

#### Benchmarking method simulation

We compare the pairwise beta-binomial method with a log-ratio transformation followed by a linear model (with and without zero imputation, LRLM and LRLM-2) and by nonparametric methods (Wilcoxon and permutation tests, named LRW and LRP). With the LRLM approach, we use the log-ratio transform ${\rm log}(Y_{i,a} / Y_{i,b}) = Y^{\prime }_i$. As with the beta-binomial model, the starting point of the LRLM approach is the reduced taxon table involving two taxa only. We then proceed to log-ratio transform the counts using ${\rm log}(Y_{i,a} / Y_{i,b}) = Y^{\prime }_i$, where $Y^{\prime }_i$ is assumed to be approximately normally distributed with constant variance *σ*^2^ and mean *μ*_*i*_. A linear regression model specified as *μ*_*i*_ = *γ*_0_ + *γ*_1_*x*_*i*_, where *γ*_0_ is the intercept representing the expected log ratio for the control group and *γ*_1_ indicates the treatment-specific difference in log ratio, can be used for testing for differential abundance. Since the transformation is not defined for values of *Y* = 0, we make use of the field standard pseudocount imputation and replace each 0 by a pseudocount of 1, where 1 represents an *ad hoc* choice (37). This approach is identical to Aitchison’s additive log-ratio transformation for a two-part composition followed by a linear model (31). Significance testing of the group difference parameter *γ*_1_ is done using a Wald test. The parameter fitted in the LRLM is an empirical, transformation-based equivalent of the odds ratio estimated in the beta-binomial regression model. LRLM-2 is identical to LRLM, except that samples where both counts are zero are removed from the analysis. The nonparametric reference methods make use of the same data transformation, with the *t*-test being replaced by either the Wilcoxon’s rank sum test (LRW) or a permutation test (LRP) (39–42).

#### Resampling-based nominal error rate assessments

To study type I error rates of the various methods under realistic data-generating scenarios, without parametric assumptions, we make use of resampling. In these analyses, we use *Bifidobacterium* and *Anaerostipes* as the denominator taxa. These taxa represent two opposites: *Bifidobacterium* is highly prevalent and usually highly abundant, while *Anaerostipes* is highly sparse with 80% zeros across the two reference studies and, if present, tends to have low counts. For each denominator taxon, we have 50 possible pairwise combinations. These situations thus represent scenarios with type I error rates to be expected for the vast majority of possible pairwise comparisons in infant microbiome data.

Since we combine data from two studies, the data for each possible taxon pair are indexed by sample and study of origin. For a given sample size *n* = 20, 40, 60, 80, 100 and 200, we sample *n*/2 samples with replacement from each study and randomly label half the samples within each study sample to either treatment or control group. For the log-ratio linear model and the beta-binomial model, we test for differences between treatment groups while controlling for study-specific effects using an additive model that symbolically reads *study* + *treatment*. Nonparametric testing is done ignoring possible heterogeneity across studies.

For the log-ratio linear model, the beta-binomial model, and both nonparametric tests, sampling and testing are repeated 10 000 times for each possible taxon pair and sample size combination. For each combination, we estimate the type I error rate of the method by recording the proportion of rejections of the null hypothesis of no difference in expectation between groups at nominal *α* = 0.05. The results represent test-wise estimates of nominal error rates for the methods at various sample sizes and biological reference data. Since testing is done on the basis of resampled data and thus not impacted by any parametric distributional assumption made, the results of the nominal error rate evaluations are not favoring any method in particular.

#### Parametric simulation-based power and coverage assessment

Power curve estimation requires parametric assumptions and a gradient of increasing effect sizes for evaluation. The parametric simulations are based on the beta-binomial model and, as in the resampling, we use *Bifidobacterium* and *Anaerostipes* as the denominator taxa. We use one dispersion estimate per denominator. For each denominator, we fit a beta-binomial regression model to each of the 50 pairs, while accounting for possible reference study effects. We then take the median dispersion (across 50 pairs/ratios) per denominator (0.46 for *Bifidobacterium* and 5.39 for *Anaerostipes*).

When generating count data, we need to provide count values for the pairwise sampling effort (i.e. pairwise totals). To mimic the data closely, we sample pairwise totals from the empirical pairwise totals of the 50 possible pairwise taxon combinations with *Bifidobacterium* and *Anaerostipes* as the denominator. Median pairwise totals for taxon pairs with *Bifidobacterium* as the denominator range from 9610 to 12 954. With *Anaerostipes* as denominator, the median pairwise totals range from 0 to 9684. Note that this implies that beta-binomial data can be generated using a set total of zero, leading to zero counts for both numerator and denominator taxa regardless of parameter values. In the beta-binomial model, such pairs do not provide viable information and are removed from analysis, while the log-ratio transformation-based methods deal with such samples using pseudocount imputation.

In order to provide an interpretable range of possible effect sizes, we fix the control group odds to ${1}/{1}$, ${1}/{9}$, ${1}/{99}$ and ${1}/{999}$. This represents pairwise probabilities for the numerator taxon of 0.5, 0.1, 0.01 and 0.001, respectively. As with the resampling, the evaluated sample sizes are *n* = 20, 40, 60, 80, 100 and 200. Combined with four values for the control group odds, this gives 24 scenarios, per dispersion. Each scenario is extended to create a power curve as follows: We increase the expected success probability of the numerator in the treatment group by steps of 0.05 until we reach an absolute difference in proportion of 0.45. The resulting range of proportions serves as expectations for a treatment group that can be compared with the proportions of the control group. In the scenarios inspired by *Bifidobacterium*, the power is additionally evaluated using increments of 0.005 (instead of 0.05). We also include a difference in proportion of 0, giving a parametric estimate of the type I error. Note that while we change the expected value across groups, we keep the dispersion fixed in order to assess method capacity to show differences under realistic levels of variability. Power is computed as the percentage of null hypothesis rejections for each sample size and baseline versus alternative model combination. Each combination is repeated 10 000 times for accurate power estimates.

We additionally use the described models to assess the coverage of the $95\%$ confidence interval around the treatment effect. Here, we compute the expected difference in proportion across the two groups and assess whether this true expected difference is captured within the computed $95\%$ confidence interval.

## RESULTS

In the data example, we see that in all three comparisons the pairwise beta-binomial is able to identify ratios that are significant (classified as FDR < 0.05). For the comparison of treatments 1, 2 and 3 versus treatment 4, we find, respectively, 6, 107 and 100 significant ratios. As is evident in Figure 3, these significant ratios are clustered among a limited number of taxa. With treatment 1, all six ratios share the same taxon. With treatment 2, a set of eight taxa is involved in all 107 ratios. With treatment 3, a set of six taxa is involved in 98 out of 100 ratios. When comparing these sets of taxa to the sets identified by the MWW method, we see a high similarity (Figure 3). With treatment 1, the MWW method identifies the same single taxon. With treatment 2, the MWW method identifies eight taxa, of which seven overlap with the proposed pairwise method. With treatment 3, the MWW method identifies four taxa that all overlap. Although we do not know the truth on which taxa have shifted, these findings demonstrate that the pairwise beta-binomial approach provides results that are consistent with the MWW method, despite their different philosophies. Note that there is no guarantee for this degree of overlap, as demonstrated in Supplement D where we illustrate that the MWW method can be misled by compositional artifacts. In the comparison with treatment 2, for example, we see that taxon 10 is identified by the MWW method. However, based on insights from the pairwise beta-binomial model, this may be the result of a compositional artifact.

**Figure 4.**
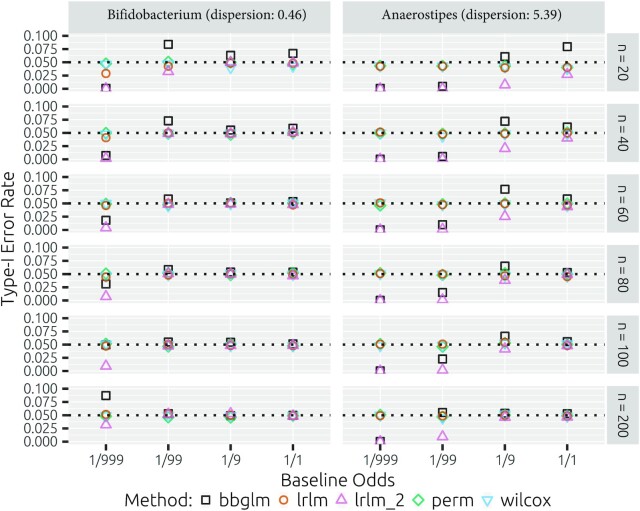
Method type I error rates for parametric null scenarios inspired by *Bifidobacterium* and *Anaerostipes*. Shown are the fraction of rejected hypotheses (*α* = 0.05). The following methods are included: the likelihood ratio test for the beta-binomial model (bbglm), the pseudocount imputation followed by log-ratio transformation linear model with *t*-test on difference parameter (lrlm), the equivalent to the previous but with omission of any zero-containing samples (lrlm_2), the imputation and log-ratio transformation-based permutation (perm) and Wilcoxon rank sum tests (wilcox).

With the parametric simulations, we assess the type I error using the 24 scenarios, inspired by *Bifidobacterium* and *Anaerostipes*, that contain no difference between the treatments (odds ratio = 1; Figure 4). At smaller sample sizes of 20 and 40 for the scenarios inspired by *Bifidobacterium*, we observe liberalness (i.e. type I error rate too large) and conservativeness (i.e. type I error rate too small) for the beta-binomial model and the LRLM without imputation. For larger odds (${1}/{99}$, ${1}/{9}$ and ${1}/{1}$), all methods’ type I error rates appear stable at sample sizes of 60 and higher. For scenarios inspired by *Anaerostipes*, we observe liberalness for the analysis with the beta-binomial model for odds of ${1}/{9}$ and ${1}/{1}$ for sample sizes up to 100. Moreover, we observe conservativeness for the beta-binomial model and the LRLM without imputation at low odds (${1}/{999}$ and ${1}/{99}$) for sample sizes up to 100. The beta-binomial model type I error rates appear to stabilize at a sample size of 200 for the ${1}/{99}$ odds scenario, while conservativeness is maintained for the ${1}/{999}$ odds scenario. The LRLM, permutation tests and Mann–Whitney–Wilcoxon tests have well-behaved type I error rates across all scenarios with sample sizes >20. Overall, no method exceeds type I error rates of 0.1 for any parametric scenario.

**Figure 5.**
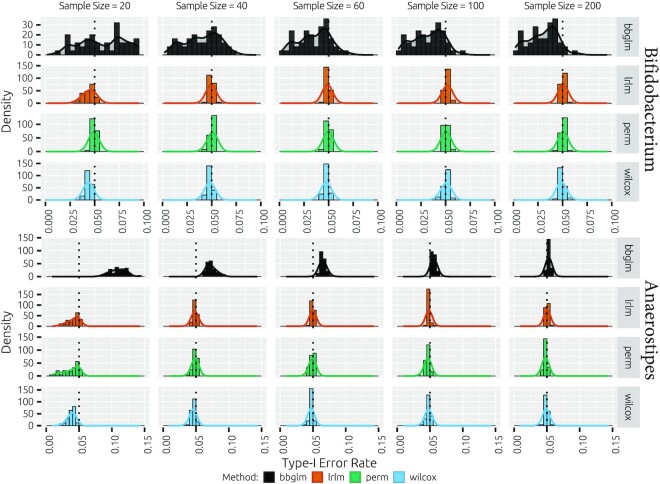
Method type I error rates for the *Bifidobacterium*- and *Anaerostipes*-inspired scenarios (50 taxon pairs each). Per pair real pairwise count data are sampled 10 000 times with various sample sizes and random treatment assignment. The type I error rate is computed as the fraction of rejections (*α* = 0.05). The following methods are included: the likelihood ratio test for the beta-binomial model (bbglm), the pseudocount imputation followed by log-ratio transformation linear model with *t*-test on difference parameter (lrlm), the imputation and log-ratio transformation-based permutation (perm) and Wilcoxon rank sum tests (wilcox). Shown are histograms and density plots (Gaussian kernel, density determines *y*-axis scale) over the 50 pairs for each sample size and method combination.

With the resampling, we similarly observe that all transformation-based methods assessed have a well-behaved type I error rate across all sample sizes >20. A sample size of 20 leads to conservativeness in both the *Anaerostipes*-based ratios and, to a lesser extent, the *Bifidobacterium*-based ratios (Figure 5). The beta-binomial model type I error rates are much more variable and display liberalness for *Anaerostipes*-based simulations from sample sizes of 20–100. At sample size of 20, type I error rate may approach values of up to 0.15. It should be noted that sample sizes here are somewhat misleading for the beta-binomial model since taxa may be pairwise absent and hence lead to many pairwise zero count samples that result in lower effective sample sizes. In contrast, the transformation-based approaches are presented with the exact sample sizes provided, albeit with large fractions of pseudocount-only samples. In the resampling simulations based on *Bifidobacterium*, there are no pairwise zeros and we instead observe variable type I error rates with increasing conservativeness for increasing sample sizes.

**Figure 6.**
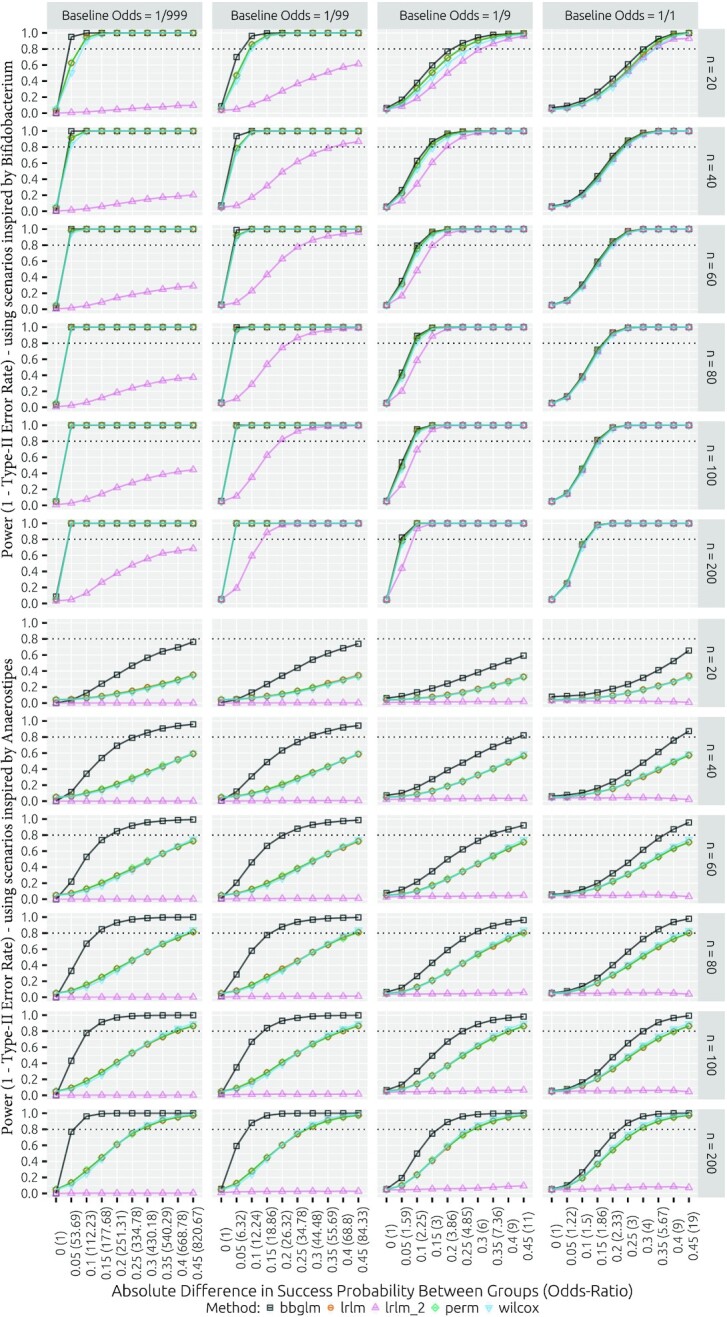
Method power for various parametric scenarios inspired by *Bifidobacterium* (top half) and by *Anaerostipes* (bottom half). The power is computed as the fraction of rejections (*α* = 0.05). The following methods are included: the likelihood ratio test for the beta-binomial model (bbglm), the pseudocount imputation followed by log-ratio transformation linear model with *t*-test on difference parameter (lrlm), the equivalent to the previous but with omission of any zero-containing samples (lrlm_2), the imputation and log-ratio transformation-based permutation (perm) and the Wilcoxon rank sum tests (wilcox). Note that while *y*-axes are comparable, each column implies vastly different odds ratios for each difference in proportion between groups.

The parametric power simulations also cover two situations: abundant taxa are represented by the scenarios inspired by *Bifidobacterium* and sparse taxa are represented by the scenarios inspired by *Anaerostipes*. In our discussion, we focus on the sample size of 60 (Figure 6 and Supplementary Figure S1 in Supplement C). The sample size of 60 is interesting since it represents a generally feasible sample size for most studies and corresponds to the sample size for which type I error rates are mostly stabilized. In the *Bifidobacterium*-inspired scenarios, the beta-binomial model achieves 80% power for odds ratios between 11 and 16 (Δ*p* = 0.01–0.015; Figure 6 and Supplementary Figure S1 in Supplement C). In comparison, the transformation-based methods require odds ratios of 31 (Δ*p* = 0.03) to achieve equivalent power (Figure 6). In the low odds scenario inspired by *Anaerostipes*, the beta-binomial model requires an odds ratio of around 250 (Δ*p* = 0.2) to achieve 80% power. Transformation-based approaches fail to reach the 80% power level for all simulated odds ratios up to 820 (Δ*p* = 0.45). For control group odds of ${1}/{99}$ in the *Bifidobacterium*-inspired scenarios, differences in power between methods are less pronounced. The beta-binomial model reaches 80% power at an odds ratio of 4 (Δ*p* = 0.03) and the transformation-based approaches reach equivalent power at odds ratios of 5.2 (Δ*p* = 0.04). At ${1}/{99}$ odds, the difference between methods remains more pronounced for *Anaerostipes*-inspired simulations. The beta-binomial model reaches 80% power at an odds ratio of 26 (Δ*p* = 0.2) and the transformation-based approaches fail to achieve equivalent power at odds ratios of 84 (Δ*p* = 0.45). For larger odds of ${1}/{9}$ and ${1}/{1}$ and *Bifidobacterium*-inspired scenarios, we observe very similar method performance with 80% power reached at odds ratios of 2.25 (Δ*p* = 0.1) and 2.33 (Δ*p* = 0.2) for the two respective baseline odds. More sizable discrepancies between methods arise again in simulations using the *Anaerostipes*-inspired scenarios, where the beta-binomial model reaches 80% power for odds ratios of 7.36 (Δ*p* = 0.35) and 9 (Δ*p* = 0.4) for control group odds of ${1}/{9}$ and ${1}/{1}$, respectively. Here, all transformation-based approaches fail to reach 80% power for the largest simulated odds ratios of 11 (Δ*p* = 0.45) and 19 (Δ*p* = 0.45). Overall, beta-binomial model power is consistently superior to that of transformation-based approaches, with the advantage being especially pronounced for sparse scenarios and more subtle for nonsparse ${1}/{9}$.

For 95% confidence interval coverage evaluations in the parametric simulations, we observe two trends. First, for both the beta-binomial model and the LRLM the 95% confidence interval coverage appears to cover null scenarios well, in line with good type I error rate performance in most cases (Figure S2 in Supplement C). Second, while the beta-binomial models show excellent effect recovery capability across all scenarios, the log-ratio linear model suffers from heavy biases in a highly situation-dependent fashion (Figure S3 in Supplement C). Low odds, high dispersion and the choice of the scenario affect confidence interval coverage heavily, with larger sample sizes decreasing coverage probabilities as expected in a biased model. The omission of pseudocount imputation only partially ameliorates this situation in some scenarios and often leads to complete model breakdown or complete loss of power.

## DISCUSSION

This paper introduces a novel method for analyzing infant microbiome data. Microbiome data from the developing infant are highly heterogeneous (8) and challenging to analyze because of their extreme variability and sparsity. The focus of the proposed method is on the ratios of the taxa, which we analyze with the beta-binomial model. We evaluate the proposed method with a data example and an extensive simulation study.

Differential abundance analysis methods in microbiome science fall into two broad categories. The first category of approaches incorporate some form of internal data normalization that aims to make statements about the absolute abundances of single taxa possible. These approaches require strong assumptions such as positing the existence of a vast majority of nondifferential taxa that are not affected by treatments or covariates (14,15). These assumptions tend not to be verifiable on the basis of sequencing data alone, but the analyses will critically rely on their validity (30,43). Commonly used analysis tools (17,18,44,45) make use of a variety of such techniques. For the data we consider in this paper, the required normalization assumptions are unlikely to be met. Similarly, proportions, total sum scaling and rarefaction can also be seen as a normalization. These methods only provide inferences on the absolute abundance if the total loads are equivalent between samples, which is unlikely for the data we consider. A second category of approaches, from the field of compositional data analyses, aim for compositional statements, which leads to inferences on ratios of taxa (16). In this paper, we propose a method based on the second approach; i.e. each taxon’s abundance is analyzed relative to some other taxon’s abundance. The advantage is that we do not need to normalize the data, and we thus do not require the normalization assumptions. Another advantage is that the method is subcompositionally coherent, meaning that the size of the full composition and the existence of other components do not impact the interpretation of the individual pairwise comparison. Correspondingly, this means that to compare quantitative measurements for new samples only the two taxa in question need to be quantified rather than the full compositions.

Simulations show that the proposed method performs well in the *Bifidobacterium*-inspired scenarios, where it is possible to detect small changes in pairwise proportion across treatment groups; the same is true for competing methods. Simulations inspired by *Anaerostipes* show diminished power for all methods and larger discrepancies between methods. In this setting, the beta-binomial model has a strong advantage over competing (transformation-based) methods. Here, for most scenarios, the beta-binomial model is the only method reaching adequate power. Deficiencies in power for the transformation-based methods are likely due to an increasing impact of pseudocount imputation and the resulting distortions of the data. The latter also affects the 95% confidence interval coverage rates in rather unpredictable ways. Here, the beta-binomial model has the advantage that it does not require *ad hoc* pseudocount imputations, resulting in good coverage for the confidence intervals. In terms of type I error, all transformation-based methods have well-behaved type I error rates across the parametric and resampling scenarios. The beta-binomial model with sparse taxa requires a larger sample size to avoid liberal type I error rates. This liberalness is likely caused by a low effective sample size, which in turn is caused by a large number of pairwise absences. Note that the method of zero imputation may influence the performance of the LRM methods, but a full evaluation of zero imputation methods (46) is beyond the scope of this article.

A full pairwise ratio analysis yields an exhaustive inference on the data (as demonstrated in the example), but practical data considerations may limit such analyses in practice. The alternative is to use a subset of taxa, or a subset of ratios. A variety of possible pairwise ratios are possible, each representing a different relationship between taxa. However, the interpretation of each ratio and its utility for inference is unique and the choice of ratios may impact any conclusions drawn. Note that if the analyses are done using one selected taxon as the denominator (the equivalent of the additive log ratio), conclusions may depend on this choice, even though using a different denominator taxon gives a statistically identical reparameterization (31,47). The question of which ratios to analyze, and how many, also depends on the inferential aim. In exploratory research, analyzing all ratios is a good option. Alternatives are to use some preselected taxon as the denominator or to use taxon pair selection (48).

As for the sparsity of the data, there are no clear rules on how to deal with this. Learning from data is only possible if there are data, and sparse taxa do not provide any relative information with respect to other taxa over most samples. Also, each sparse taxon added to a pairwise analysis will add increasingly many (underpowered) sparse comparisons to the analysis, which in turn adds substantially to the multiplicity burden. The combinations of low power, possibly elevated type I error rates and the fundamental difficulties in securing model and data trustworthiness for sparse taxa are all reasons to favor stringent data filtering steps. Although filtering is recommended, more research is needed to study the impact of such techniques on results (49–51). Sparse taxa can also be amalgamated; the resulting amalgamation is less subject to sparsity (32,48). Which taxa to include into an amalgamation and the resulting interpretation should be treated with care; haphazard combinations may lack biological relevance.

The number of ratios analyzed and equivalently the number of tests can be large. This means some form of multiple testing that corrects across all tested ratios is needed (52–54); in the example, we used the commonly applied Benjamini–Hochberg (36). Possibly more power can be gained with a method that is more adapted to our application, for example, by taking into account the structure underlying the ratios (i.e. they share taxa). This, however, requires more research. One way of reducing the multiplicity burden is to preselect ratios, either on abundance or on (*a priori* defined) biological plausibility.

## CONCLUSION

In this paper, we propose a pairwise ratio approach to analyze 16S microbial sequencing data that contain high sparsity, extreme dispersion and nonstandardizable compositionality. This approach is defined by refocusing the beta-binomial model to the pairwise perspective, rendering analysis results comparable to standard log-ratio analyses based on pairs of taxa. We compare the performance of the proposed method to a number of alternative pairwise methods. The beta-binomial model has more power in all settings, but displays some degree of type I error rate liberalness in sparse settings. In practical application, we suggest to combine the proposed method with pragmatic data reduction approaches (i.e. filtering and amalgamation), in order to maintain high power and to avoid sparse taxon pairs whose analysis depends heavily on zero modeling assumptions.

## DATA AVAILABILITY

The data used in this article are available in Supplement D.

## Supplementary Material

lqad001_Supplemental_Files
