# Supplementary figures and images for: Pairwise ratio-based differential abundance analysis of infant microbiome 16S sequencing data
Source: NAR Genom Bioinform. 2023 Jan 20;5(1):lqad001. doi: 10.1093/nargab/lqad001 (PMC9853100; doi:10.1093/nargab/lqad001)

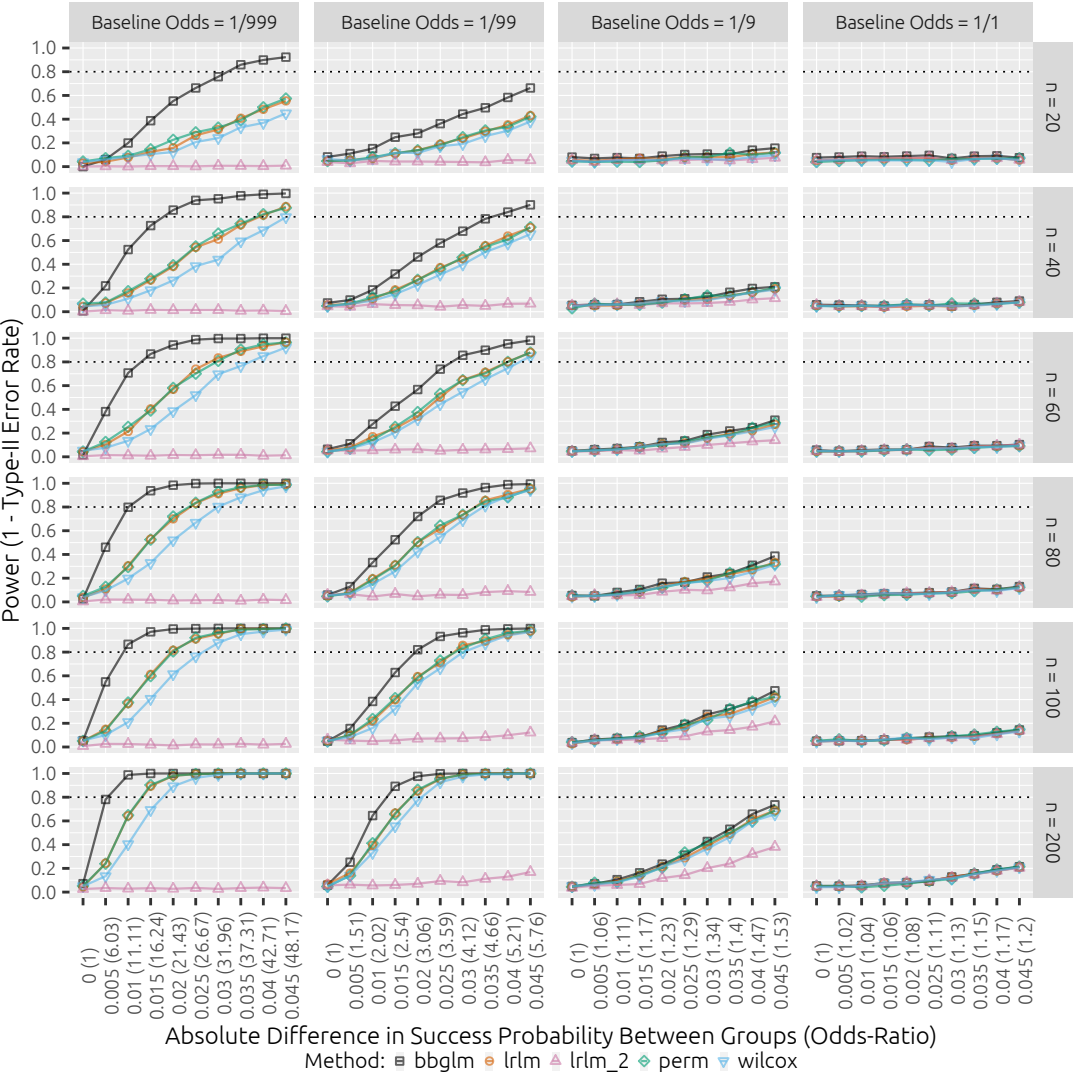

Supplement: lqad001_Supplemental_Files [file lqad001_supplemental_files.zip › figS1 parametric-power-bifido-ext-median-dispersion.pdf]

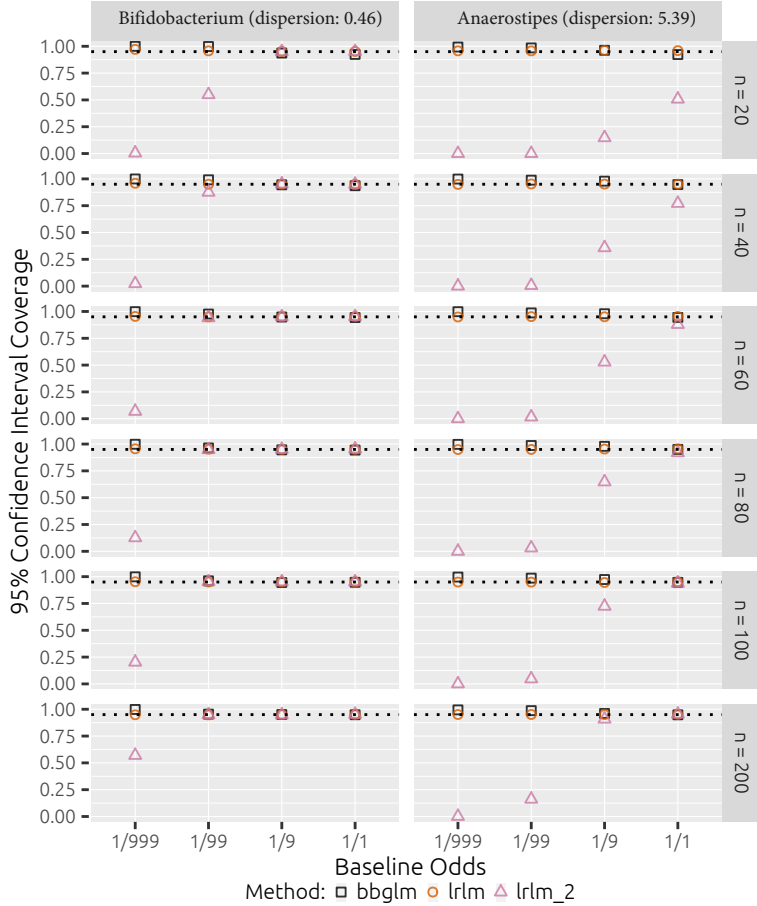

Supplement: lqad001_Supplemental_Files [file lqad001_supplemental_files.zip › figS2 combined parametric-coverage-null-scenario.pdf]

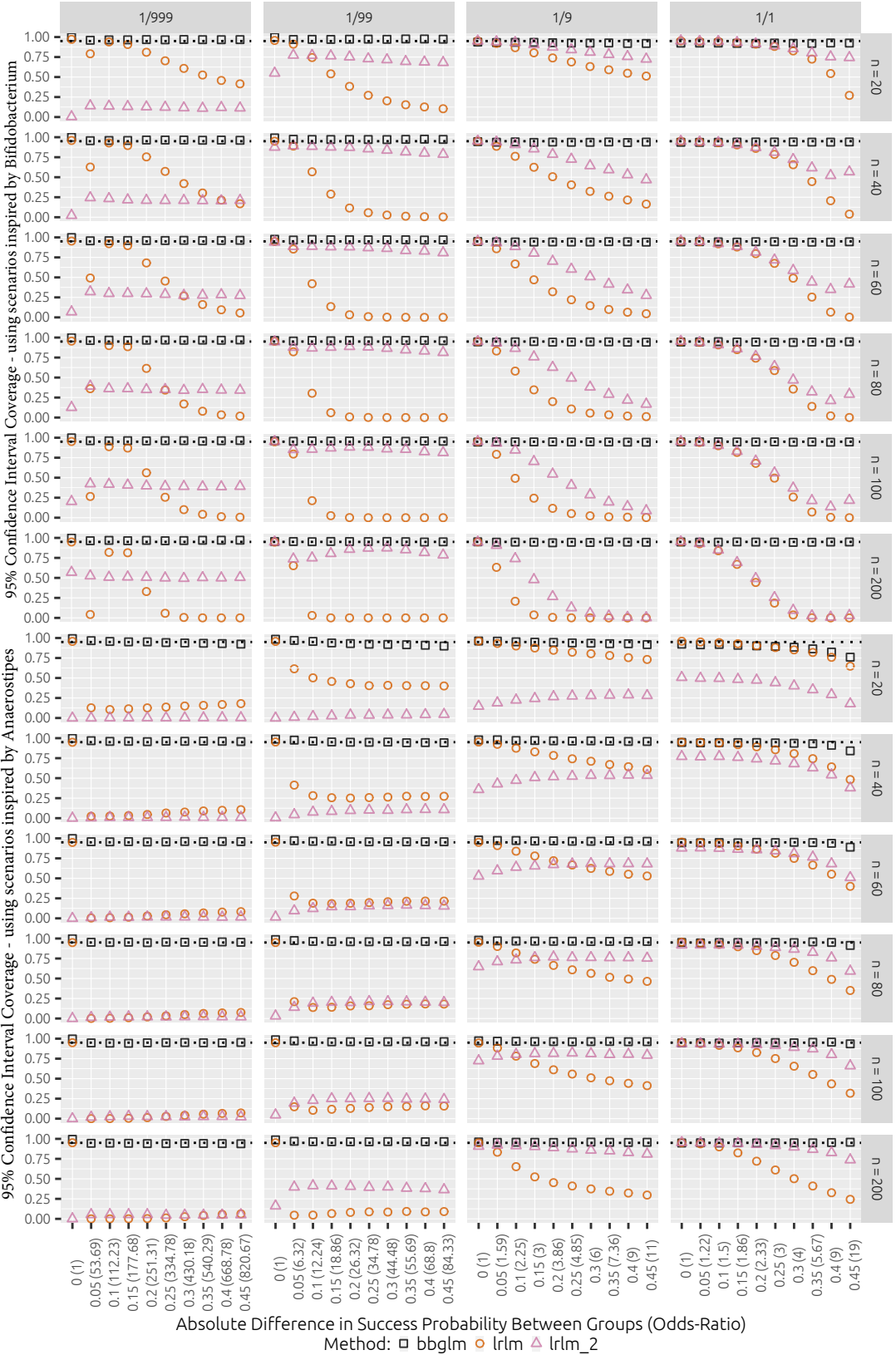

Supplement: lqad001_Supplemental_Files [file lqad001_supplemental_files.zip › figS3 parametric-coverage-scenarios.pdf]
