# Supplementary material for: Pairwise ratio-based differential abundance analysis of infant microbiome 16S sequencing data
Source: NAR Genom Bioinform. 2023 Jan 20;5(1):lqad001. doi: 10.1093/nargab/lqad001 (PMC9853100; doi:10.1093/nargab/lqad001)
Supplement: lqad001_Supplemental_Files [file lqad001_supplemental_files.zip › supplement.zip!/supplement/data example/analysis/dataExample.pdf]

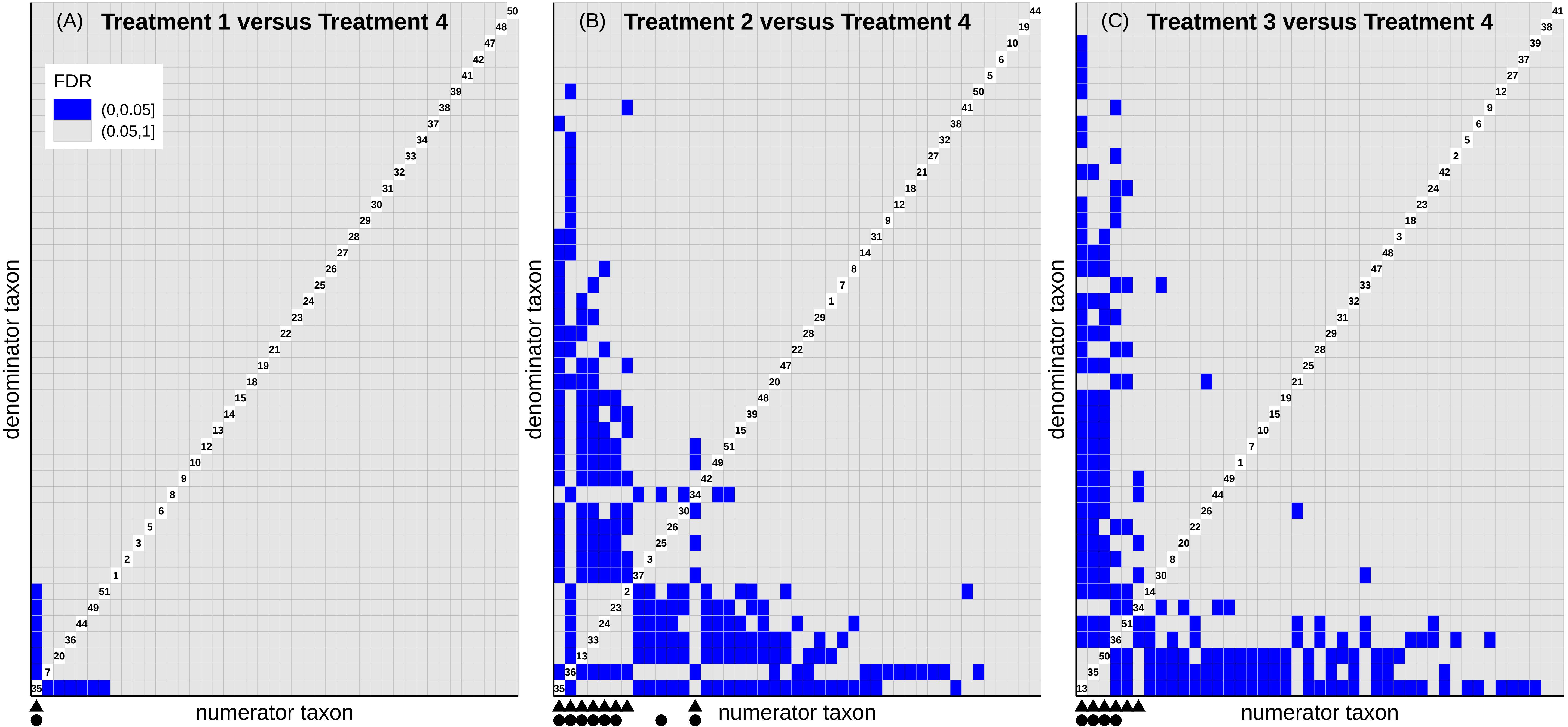

(A) Treatment 1 versus Treatment 4
(B) Treatment 2 versus Treatment 4
(C) Treatment 3 versus Treatment 4
FDR
(0,0.05]
(0.05,1]
denominator taxon
numerator taxon
