# Supplementary material for: Pairwise ratio-based differential abundance analysis of infant microbiome 16S sequencing data
Source: NAR Genom Bioinform. 2023 Jan 20;5(1):lqad001. doi: 10.1093/nargab/lqad001 (PMC9853100; doi:10.1093/nargab/lqad001)
Supplement: lqad001_Supplemental_Files [file lqad001_supplemental_files.zip › supplement.zip!/supplement/data example/figureData/figureData.pdf]

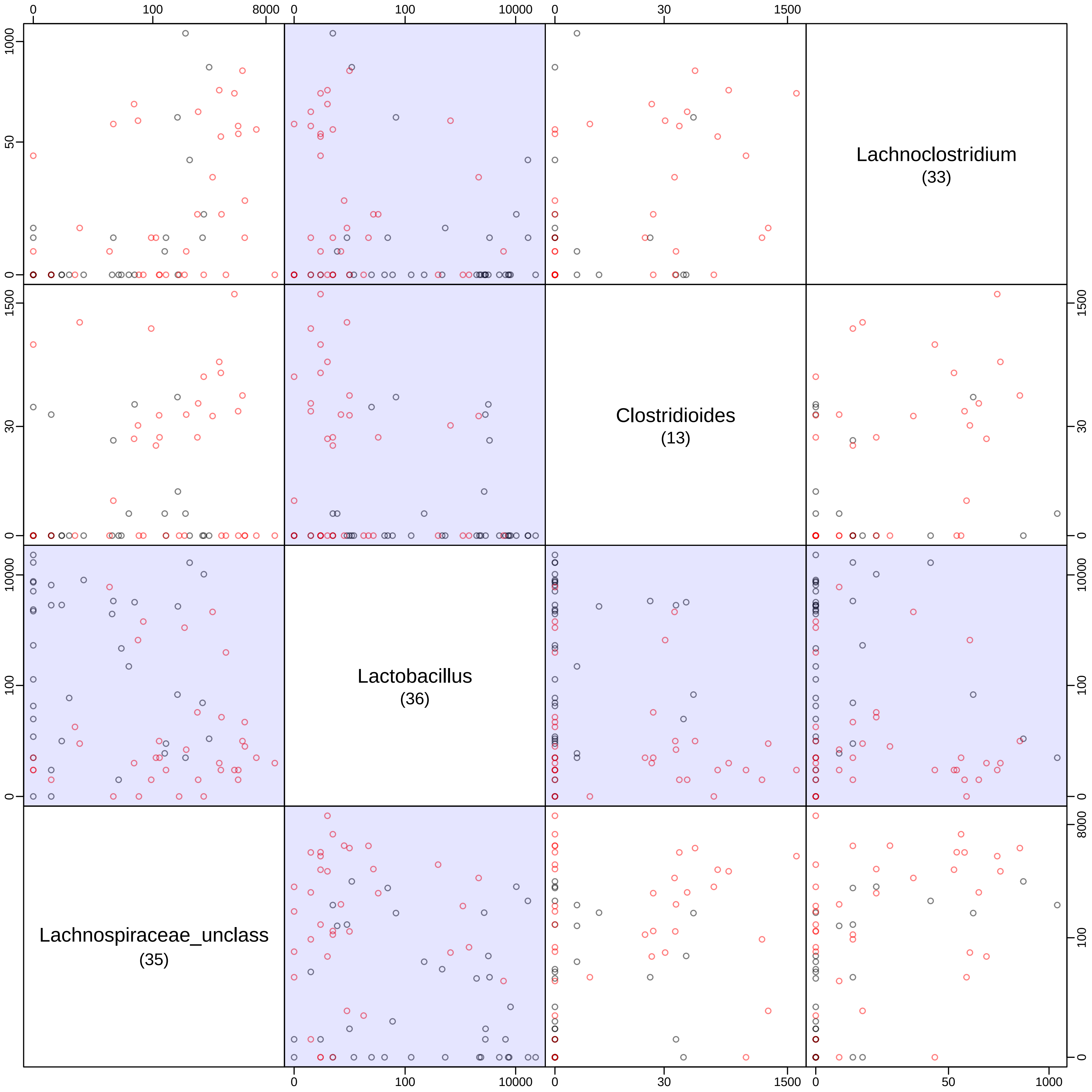

Lachnoclostridium
(33)
Clostridioides
(13)
Lactobacillus
(36)
Lachnospiraceae_unclass
(35)
0
100
8000
0
100
10000
0
30
1500
0
50
1000
