# Supplementary material for: Pairwise ratio-based differential abundance analysis of infant microbiome 16S sequencing data
Source: NAR Genom Bioinform. 2023 Jan 20;5(1):lqad001. doi: 10.1093/nargab/lqad001 (PMC9853100; doi:10.1093/nargab/lqad001)
Supplement: lqad001_Supplemental_Files [file lqad001_supplemental_files.zip › supplement.zip!/supplement/supplemental example/supplement-example.pdf]

Supplemental material: Comparison beta-binomial models

Dennis te Beest

24-9-2021

Introduction

This is an example to compare the pairwise beta-binomial (introduced in this paper) to the beta-binomial model of Martin et al. (2020); the latter we will refer to as the MWW method. Both methods are based on the beta-binomial model; the difference is that the MWW method analyses abundances relative to the total library size and the pairwise model analyses taxa relative to each other (i.e. ratios). It is well known that an analysis that focuses on abundances relative to the total library size is sensitive to what is often referred to as the "compositional bias". With "compositional bias" we refer to the effect that if some taxa increase in their abundance, all other taxa (by definition) decrease in their relative abundance, this is result of the unit-sum constraint (Aitchison, 2003). In the context of differential abundance analysis this means that if a treatment increase the abundance of one taxon, it decreases the relative abundance of all other taxa. This supplement provides an example of this bias and it demonstrates how it influences the analysis of relative abundances and that this bias does not affect an approach based on ratios. Note that the setup of the simulation is designed to display the weakness of analyzing relative abundances when the compositional bias is large. In practice, this bias may be smaller, but generally its size is unknown.

Simulation setup

This supplemental example is based on the data example of the main document, i.e. we use the same 149 samples and the same 51 taxa. Based on these data we conduct a simulation study. Per simulation we randomly select 75 samples that are assigned to one treatment group. The remaining 74 samples are assigned to a second treatment group. For the first treatment group two taxa receive a differential shift by multiplying them by 8 (i.e. an 8-fold change). The remaining 49 taxa remain unchanged for both groups. For both methods we then follow (per simulation) the same approach as in the data example of the main document. For the MWW method we test each taxon separately and select taxa with an FDR (Benjamini-Hochberg) below 0.05. For the the pairwise beta-binomial method we test each ratio separately (i.e. 1275 ratios for 51 taxa) and select all ratios with an FDR (Benjamini-Hochberg) below 0.05. We then asses how often the two shifted taxa are involved in the selected ratios.

Number of selected taxa and ratios

The barplot below displays the frequencies at which taxa have been selected with the MWW method. The red bars indicate the two taxa that have been shifted. The shifted taxa are identified in most simulations, but it is also clear that a large number of taxa are incorrectly identified. In total across 50 simulations 331 taxa are identified, of these 238 are incorrect and 93 are correct, this equates a false discovery rate of 0.72.

With the pairwise ratio beta-binomial model we identify 359 ratios across 50 simulations, of which 342 ratios contain either of the shifted taxa. This corresponds a false discovery rate 0.05, which corresponds to chosen level for the Benjamini-Hochberg correction (0.05).

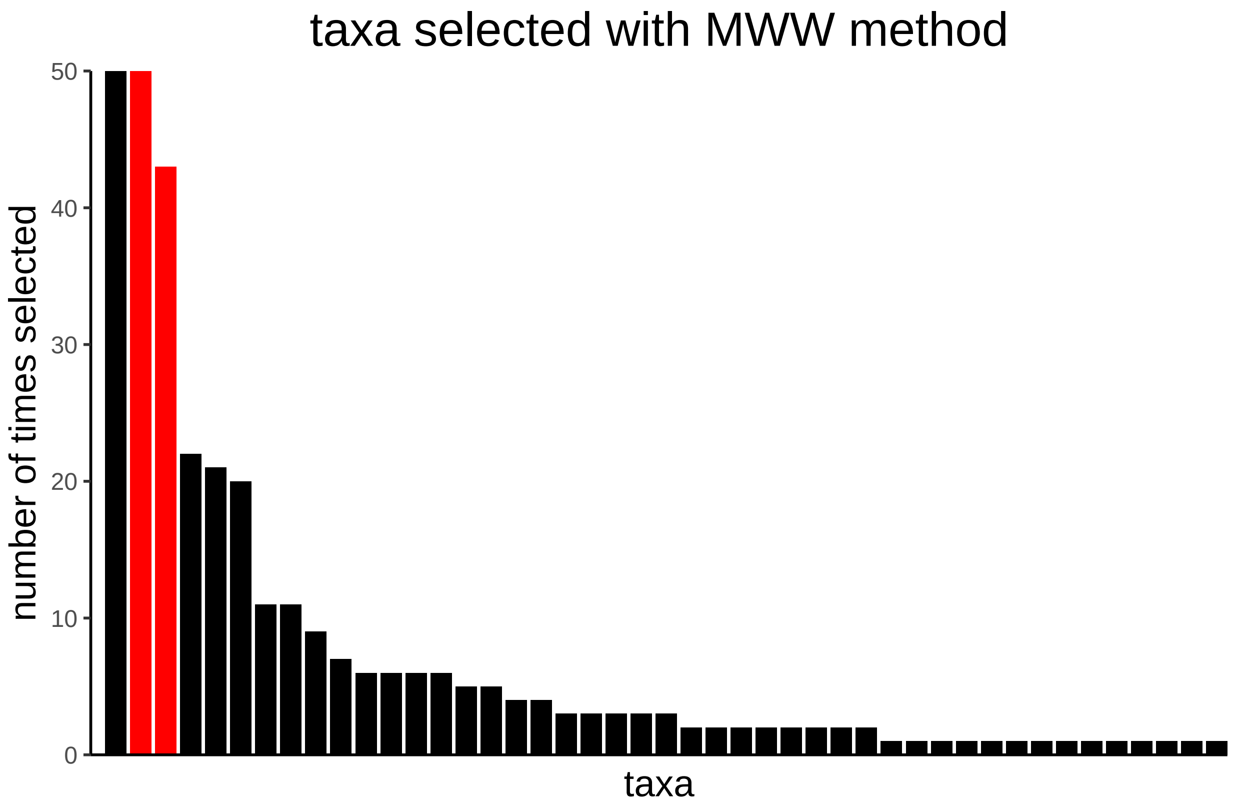

Figure 1. Number times taxa are selected with MWW method across 50 simulations

Examining the estimates

The reason why the MWW method performs so poorly in this example is better understood when we examine the estimates of the effect sizes. Figure 2 below visualizes the estimates across all simulations. In this figure it is clear that the estimates of the MWW method are centered around -0.25. We also see a long tail on the right; this tail contains mostly estimates that belong to the two shifted taxa. The estimates in the left tail belong to taxa that have not received a differential shift, but nevertheless have absolute values that are equal to those in the right tail (as a result of the shift in their relative abundance). Some of these values can become so low that they are easily declared significant (i.e. we test against a null hypothesis of "no effect"). With the ratio method the estimates are centered around zero. The significant ratios cause a somewhat thicker tails on both sides of the distribution. This corresponds with what is expected given the simulation setup.

Discussion

Although the result of this supplement may be a surprise to some, note that these results are well-known and well-described, particular in the field of compositional data analysis (Aitchison, 2003; Morton et al., 2019). The simulation is designed to display the weakness of analyzing relative abundances when the compositional bias is large. The fold change of 8 is selected because it illustrates the compositional bias clearly. Reducing the fold change, reduces the effect of compositional bias, and as a result less taxa without shift are pushed over the FDR significance threshold with the MWW method (and reducing the number of false discoveries). Although this differential shift is a relatively large, it can be reasonably expected in some microbiome studies. As demonstrated, ratio methods are insensitive to this bias, as is known from theory and this is by design (Aitchison, 2003; Morton et al., 2019).

References

MARTIN, B. D., WITTEN, D. and WILLIS, A. D. (2020). Modeling microbial abundances and dysbiosis with beta-binomial regression. Ann. Appl. Stat. 14 94–115.

AITCHISON, J. (2003). The statistical analysis of compositional data. Blackburn Press, Caldwell, N.J.

MORTON, J. T., MAROTZ, C., WASHBURNE, A., SILVERMAN, J., ZARAMELA, L. S., EDLUND, A., ZENGLER, K. and KNIGHT, R. (2019). Establishing microbial composition measurement standards with reference frames. Nature Communications 10 2719

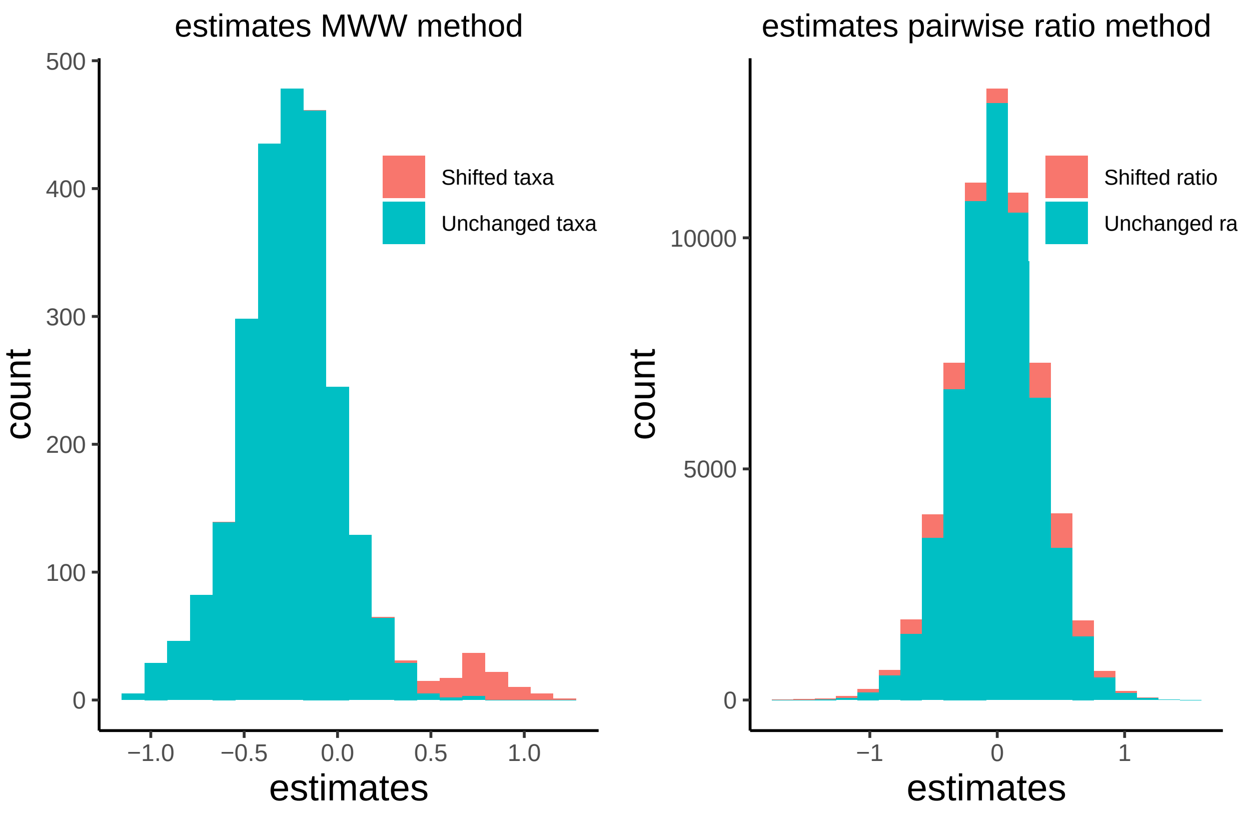

Figure 2. Estimated effect sizes across 50 simulations
